# Metabolic abnormalities in adult T-cell leukemia/lymphoma and induction of specific leukemic cell death using photodynamic therapy

**DOI:** 10.1038/s41598-018-33175-7

**Published:** 2018-10-08

**Authors:** Takashi Oka, Hajime Mizuno, Masumi Sakata, Hirofumi Fujita, Tadashi Yoshino, Yoshihisa Yamano, Kozo Utsumi, Tsutomu Masujima, Atae Utsunomiya

**Affiliations:** 1 Department of Pathology and Oncology, Graduate School of Medicine, Dentistry and Pharmaceutical Sciences, Okayama University, Okayama, 700-8558 Japan; 2 Department of Virology, Graduate School of Medicine, Dentistry and Pharmaceutical Sciences, Okayama University, Okayama, 700-8558 Japan; 3 Laboratory for Single Cell Mass Spectrometry, RIKEN Quantitative Biology Center (QBiC), Osaka, 565-0874 Japan; 4 Department of Cytology and Histology, Graduate School of Medicine, Dentistry and Pharmaceutical Sciences, Okayama University, Okayama, 700-8558 Japan; 5 Department of Rare Diseases Research, Institute of Medical Science, St. Marianna University School of Medicine, Kawasaki, 216-8512 Japan; 6 Department of Hematology, Imamura General Hospital, Kagoshima, 890-0064 Japan; 7 Present Address: Laboratory of Analytical and Bio-Analytical Chemistry, School of Pharmaceutical Sciences, University of Shizuoka, Shizuoka, 422-8526 Japan

## Abstract

Adult T-cell leukemia/lymphoma (ATL) is an aggressive T-cell neoplasm caused by human T-cell leukemia virus type I (HTLV-I). Therapeutic interventions have not been associated with satisfactory outcomes. We showed that the porphyrin metabolic pathway preferentially accumulates the endogenous photosensitive metabolite, protoporphyrin IX (PpIX) in ATL, after a short-term culture with 5-aminolevulinic acid (ALA). PpIX accumulated 10–100-fold more in ATL leukemic cells when compared to healthy peripheral blood mononuclear cells (PBMCs). Patient specimens showed dynamic changes in flow cytometry profiles during the onset and progression of ATL. Furthermore, 98.7% of ATL leukemic cell death in the ATL patient specimens could be induced with 10 min of visible light exposure, while 77.5% of normal PBMCs survived. Metabolomics analyses revealed that a specific stage of the metabolic pathway progressively deteriorated with HTLV-I infection and at the onset of ATL. Therefore, this method will be useful in diagnosing and identifying high-risk HTLV-I carriers with single cell resolutions. Photodynamic therapy in the circulatory system may be a potential treatment due to its highly-specific, non-invasive, safe, simultaneous, and repeatedly-treatable modalities.

## Introduction

Adult T-cell leukemia/lymphoma (ATL) is an aggressive malignant disease of the CD4(+) T lymphocytes associated with the human T-lymphotropic virus type I (HTLV-1) infection^1–4^. Approximately 20 million individuals are infected with HTLV-1 worldwide^5^, 1.1 million of whom reside in Japan. The annual number of ATL incidences is estimated to be approximately 1,000 cases in Japan alone^6^. HTLV-1 infections, which occur mainly via breast feeding, cause ATL in 3–5% of HTLV-1 asymptomatic carriers (ACs) after a long latent period of 40–60 years. Such a long latent period suggests that a multi-step leukemogenic and/or lymphomagenic mechanism is involved in the development of ATL^7^. The diversity of the clinical features and prognosis of ATL patients has led to its classification into 4 categories based on lactate dehydrogenase (LDH), calcium values, and organ involvement: acute and lymphoma types showing aggressive phenotypes, and chronic and smoldering types showing indolent phenotypes^8,9^. HTLV-1 also causes several inflammatory diseases such as infective dermatitis, HTLV-associated Uveitis, and HTLV-1-associated myelopathy-tropical spastic paraparesis (HAM/TSP), a chronic inflammatory disease of the central nervous system (CNS) characterized by progressive spastic paraparesis, lower limb sensory disturbance, and bladder or bowel dysfunction^10–13^. HTLV-1 pathogenesis has been extensively investigated in terms of the viral regulatory proteins, HTLV-1 Tax and HTLV-1 basic leucine zipper factor (HBZ), which are supposed to play key roles in HTLV leukemogenesis/lymphomagenesis^14,15^. Recently, a large-scale genetic study delineated the entire portrait of genetic and epigenetic aberrations in ATL and identified a large number of novel mutational targets^16^. However, the detailed mechanisms triggering the onset and progression of ATL remains to be elucidated^14–18^. Therapeutic interventions, including intensive chemotherapy for aggressive ATL, are not associated with satisfactory outcomes mainly because ATL cells are often resistant to chemotherapeutic agents. Moreover, patients with ATL also frequently suffer from a number of opportunistic infections. Recently, allogeneic hematopoietic stem cell transplantations and molecular targeted therapies, including the anti-CCR4 monoclonal antibody mogamulizumab, were shown to improve overall survival in ATL patients. Although new therapeutic options are gradually improving the curability of ATL, treatments remain a challenging prospect for ATL patients^19,20^. Therefore, to improve the clinical outcomes for ATL patients, rigorous investigations and development of new therapeutic modalities are necessary to prevent ATL development in HTLV-1 asymptomatic carriers and ATL progression from indolent to aggressive types.

Photodynamic therapy (PDT) is a recently-developed anticancer treatment that utilizes the generation of singlet oxygen and other reactive oxygen species (ROS) in cancer tissues. The body’s own intrinsic, biochemical, metabolic molecules that localize within tumor tissues are used as light-activated therapeutic targets. 5-Aminolevulinic acid (5ALA) is the first metabolite in the heme biosynthesis pathway in humans. In addition to the end product heme, this pathway also produces other porphyrin metabolites. Protoporphyrin IX (PpIX) is a heme precursor porphyrin that exhibits good fluorescence and photosensitizing activity. As a natural photosensitizer, PpIX absorbs energy directly from a harmless visible light source and then transfers the energy to molecular oxygen to create an activated form of oxygen called singlet oxygen (^1^O_2_) and other reactive oxygen species (ROS). This singlet oxygen is supposed to be the real cytotoxic agent that reacts rapidly with cellular components and causes the tumor cell damage that finally leads to cell death with necrosis and/or apoptosis and tumor destruction. ALA has been investigated with respect to the detection and treatment of tumors in a number of organs. Its application as a diagnostic tool leads to the selective accumulation of the heme precursor PpIX in tumors and precancerous lesions. The clinical applications of photodynamic diagnosis (PDD) range from better definition of surgical margins in skin or brain tumors to better detection of flat precancerous lesions and early tumors in the bladder, endobronchial tissues, breast, and GI tract^21–25^. Although the clinical potential of PDT has been recognized for more than 35 years, its applications are still in the initial stages^26–28^ mainly because of the poor penetration of light into tissues more than 3 mm thick in order to induce sufficient tumor necrosis and/or apoptosis and also due to the incubation time required by ALA-PDT between drug application and light exposure in order to be metabolically converted into PpIX.

Furthermore, PDT has not been extensively investigated with clinical specimens for hematopoietic malignancies such as leukemia. In the present study, we clearly showed that PDT successfully and specifically induced ATL leukemic cell death with minimal effects in the normal peripheral blood mononuclear cells (PBMCs) of ATL patient specimens. Additionally, the dynamic changes in the flow cytometry (FCM) profiles observed during the onset of ATL clearly identified the high-risk HTLV-1 carriers who were highly susceptible to the development of ATL. Furthermore, the dynamic FCM profile changes observed during the progression of indolent ATL also clearly identified patients with high-risk indolent ATL who appeared to have developed or were likely to develop the aggressive subtypes. This suggestively implicated the preventive therapy of ATL with ALA-PDT by eliminating the small population of leukemic cells before the overt onset of ATL.

## Results

**Figure 1:**
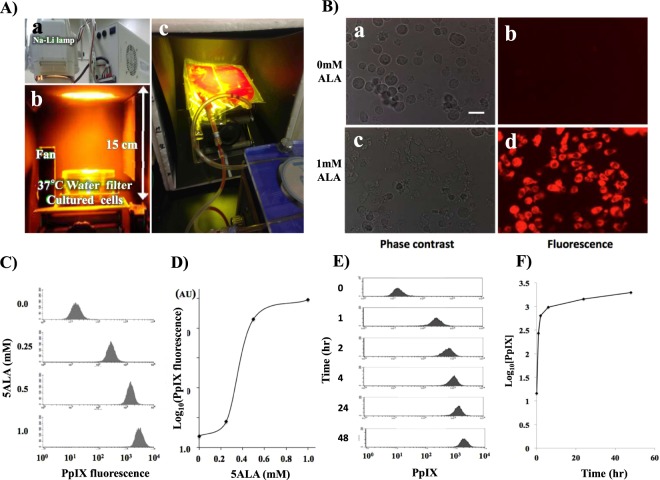
(**A**) The PDT device. (a,b) An Na-Li lamp was used as a light source along with a 37 °C water filter, a fan to prevent increases in specimen temperature. (c) For the whole blood PDT experiments, whole blood was circulated using a Perista-pump during light exposure. (**B**) Accumulation of PpIX in TLOm1 cells after incubation with 1 mM ALA. (a,b) 0 mM ALA, (c,d) 1 mM ALA, (a,c) Phase-contrast microscope, (b,d) Fluorescent microscope. Bar indicates 15 µm. Strong PpIX fluorescence in TLOm1 cells was observed only after incubation with 1 mM ALA. (**C**,**D**) Dose-response experiments. Dose response of 5ALA from 0–1.0 mM against PpIX fluorescence in TLOm1 cells. (**E**,**F**) Time course experiments. Incubation time dependence of PpIX fluorescence in TLOm1 cells after addition of 1 mM ALA to culture medium.

**Figure 2:**
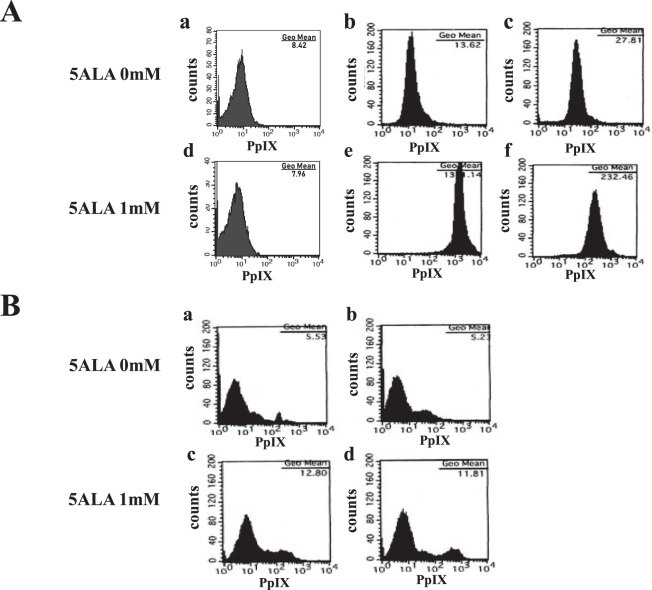
(**A**) ATL cell-specific accumulation of PpIX. Accumulation of PpIX in (a,d) healthy PBMCs, (b,e) TLOm1, and (c,f) ED40515(−) cells after incubation with (a–c) 0 mM or (d–f) 1 mM ALA for 24 h. Healthy PBMCs showed no accumulation of PpIX even in the presence of 1 mM ALA. Contrastingly, TLOm1 and ED40515(−) cell lines showed strong PpIX accumulation (almost 100-fold or 10-fold, respectively) in 1 mM ALA when compared to that in 0 mM ALA. (**B**) Accumulation of PpIX in the PBMCs of chronic dermatitis patients. Aberrant PpIX accumulation profiles in the PBMCs of chronic dermatitis patients (a,c) without stimulation and (b,d) co-stimulated with CD3/CD28, followed by incubation with (a,b) 0 mM or (c,d) 1 mM ALA. (a) In the culture containing 0 mM ALA, the PBMCs of the chronic dermatitis patient contained a weak aberrant PpIX peak (a), which was enhanced after incubation with (c) 1 mM ALA. (b) After CD3/CD28 co-stimulation, the additional aberrant PpIX peak became stronger than that in (a) the 0 mM ALA incubation. (d) The additional aberrant PpIX peak was strongest after CD3/CD28 co-stimulation in 1 mM ALA, indicating that activated non-malignant cells, such as inflammatory or artificially-stimulated cells, also accumulated PpIX; however, the levels were lesser than that in the leukemic cells.

We initially tried to determine if the specific fluorescence of PpIX could be detected in ATL leukemic cells after incubation with ALA. The ATL cell line, TLOm1, showed strong PpIX fluorescence when cells were incubated for 24 h in the presence of 1 mM ALA, whereas no fluorescence was observed in the mock standard culture without ALA (Fig. 1B). Dose response analyses of ALA against PpIX fluorescence using flow cytometry (FCM) showed PpIX fluorescence saturation in 1.0 mM ALA, which was found to be the optimum concentration for intracellular PpIX accumulation (Fig. 1C,D). Time course experiments indicated a quick accumulation of PpIX in the first 4–5 h, followed by a gradual increase towards saturation (Fig. 1E,F). Next, we confirmed if the obvious increase in PpIX accumulation in the ATL cell lines was specific using 2 ATL cell lines and healthy PBMCs. TLOm1 cells showed more than a 100-fold increase in PpIX fluorescence in the presence of 1 mM ALA when compared to the 0 mM ALA condition. Another ATL leukemic cell line, ED40515(−), exhibited an 8.4-fold increase of PpIX under the same conditions. Other types of ATL leukemic T-cells (ATL55T, ATL43Tb) and ATL derived T-cells (MT1 and HUT102) also showed strong increase in PpIX fluorescence in the presence of 1 mM ALA (Table S2). Furthermore, various type of hematopoietic malignancies, including T- and B-cell leukemia/lymphoma, myeloid leukemia and solid tumors of cancer and sarcoma also showed increase in PpIX fluorescence with different extent in the presence of 1 mM ALA (Figs S1, S2 and Table S2). PpIX fluorescence after incubation in the presence of 1 mM ALA for 48 h showed similar results as for 24 h incubation. However, healthy PBMCs showed no accumulation of PpIX even in the presence of 1 mM ALA, indicating that PpIX accumulation in the presence of 1 mM ALA was specific to malignant cells as well as ATL cells (Fig. 2A).

Interestingly, PBMCs from a dermatitis patient showed a different pattern in the FCM profiles. Aberrant PpIX peaks were observed in the presence and absence of 1 mM ALA, suggesting that some kind of activated cell populations were probably contributing to this phenomenon. To confirm this hypothesis, we used the Dynabeads^®^ Human T-Activator CD3/CD28 kit to activate and expand the T cells of the dermatitis patient PBMCs. Activated dermatitis patient PBMCs showed additional strong PpIX peaks especially in the presence of 1 mM ALA, indicating that non-malignant PBMCs also accumulated PpIX during activated conditions such as inflammation (Fig. 2B). Moreover, the CD3/CD28 T cell activation treatment against the HTLV-1 carriers and smoldering ATL PBMCs induced stronger PpIX accumulation when compared to that in healthy PBMCs, suggesting that smoldering ATL patients and carriers somehow acquired abnormalities in the porphyrin metabolic pathway (Fig. S3).

**Figure 3:**
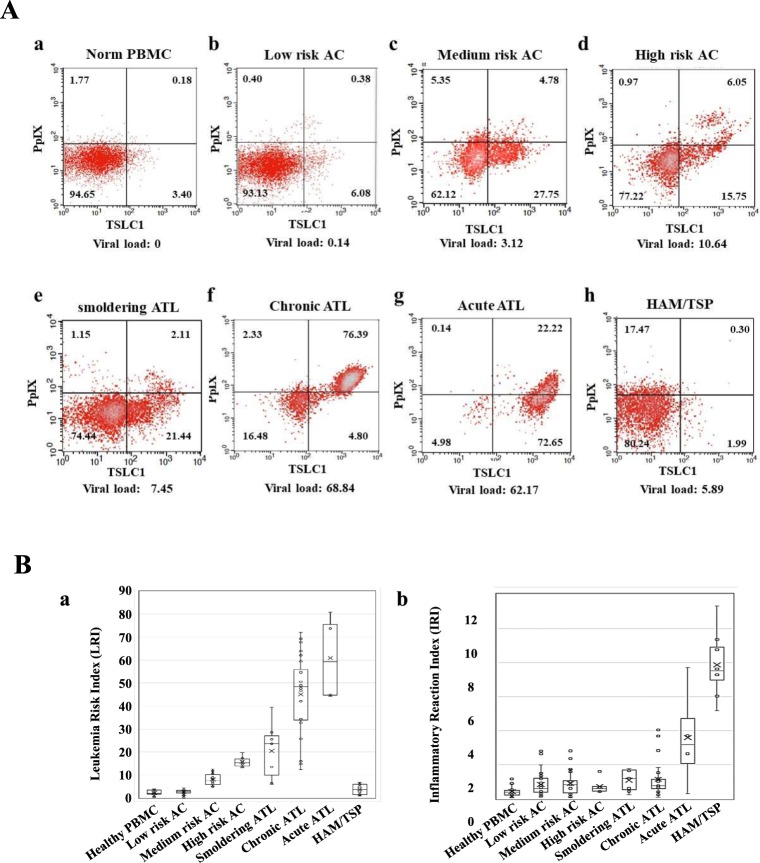
(**A**) Flow cytometry (FCM) profiles reflecting the changes in the PpIX/TSLC1 parameters during the onset and progression of ATL and HAM/TSP. Gradual changes in the PpIX/TSLC1 profiles, shifting from PpIX(−)/TSLC1(−) in (a) healthy PBMCs to (e–g) PpIX(−)/TSLC1(+) and PpIX(+)/TSLC1(+) in relation to the onset and/or progression from smoldering-, to chronic-, to acute-ATL. (b–d) AC PBMCs showing intermediate profiles. (h) HAM/TSP showing an increase in PpIX(+)^dim^/TSLC1(−) fractions when compared to those in healthy PBMCs in addition to the PpIX(−)/TSLC1(−) fraction. (**B**) Leukemia Risk Index (LRI) and Inflammatory Reaction Index (IRI) changes during the onset and progression of ATL and HAM/TSP. LRI and IRI are defined as in Table 1. (a) Onset and progression-dependent increase of LRI as observed in ATL. The HAM/TSP specimen showed almost the same LRI value as healthy PBMCs. AC PBMCs were classified to 3 categories (low-, medium-, and high-risk) according to the LRI value, which corresponded to the typical FCM profiles in (**A)**. (b) HAM/TSP showing high IRI values when compared to the other categories.

Next, PBMCs from healthy volunteers, asymptomatic HTLV-1 carriers (ACs), and patients with smoldering-, chronic-, acute-type ATL, and HAM/TSP were analyzed via FCM using PpIX and ATL leukemic cell marker: TSLC1/CADM1 (tumor suppressor in lung cancer 1/cell adhesion molecule 1) parameters, which showed that dynamic changes in the 2-D FCM profiles could be observed from smoldering-, to chronic-, to acute-type ATLs in accordance with progression (Fig. 3). In order to clarify the profile differences, Leukemia Risk Index (LRI) = 0.0001X (LL) + 0.5X (LR) + (UR) and Inflammatory Reaction Index (IRI) = 0.0001X (LL) + 0.5X (UL) were defined (Table 1). Asymptomatic HTLV-1 carrier PBMCs were classified into 3 categories according to the FCM profiles: Low-risk ACs (similar to healthy profile), LRI < 4.5; Medium-risk ACs (intermediate profile), 4.5 ≤ LRI ≤ 13.0; High-risk ACs (similar to smoldering ATL profile), 13.0 < LRI (Fig. 3Ba,b; Table 1). While a gradual increase in LRI was observed from smoldering-, to chronic-, to acute-type ATLs, HAM/TSP demonstrated an LRI almost similar to that of healthy donors. However, the IRI of HAM/TSP was high when compared to the other groups, which correlated with the distinct reactive population of the PpIX(+)^dim^/TSLC1(−) cells in the FCM profile. Furthermore, evident heterogeneities in PpIX/TSLC1 profile were observed. Especially, there are various intermediate patterns of PpIX/TSLC1 profile in chronic ATL PBMCs, indicating that many sub-population of cells show clusters having different intensity of PpIX and/or TSLC1 in individual ATL patients (Fig. S4).

**Table 1:** Average Leukemia Risk Index (LRI) and Inflammatory Reaction Index (IRI) in ATL and HAM/TSP patients.

Categories	No. of samples	Average IRI	SD of IRI	P value (IRI)*	Average LRI	SD of LRI	P value (LRI)*
Healthy PBMC	20	0.4	0.28	—	2.33	1.25	—
Low risk AC	32	0.83	0.67	0.003	2.6	0.98	0.189
Medium risk AC	23	0.91	0.71	0.006	8.15	2.47	<0.001
High risk AC	6	0.72	0.41	0.058	15.71	2.3	<0.001
smoldering ATL	7	1.08	0.61	0.034	20.41	11.48	0.008
chronic ATL	21	1.13	1.01	0.002	45.08	17.47	<0.001
acute ATL	4	3.58	2.68	0.132	60.97	16.58	0.009
HAM/TSP	10	7.86	1.81	<0.001	3.73	2.33	0.131

Inflammatory Reaction Index (IRI) = 0.0001X (LL) + 0.5X (UL).

Leukemia Risk Index (LRI) = 0.0001X (LL) + 0.5X (LR) + (UR).

LRI (Low risk AC) < 4.5, 4.5 ≦ LRI (Midium risk AC) ≦ 13.0, 13.0 < LRI (High risk AC).

LL: Lower Left, UL: Upper Left, UR: Upper Right and LR: Lower Right of Quadrant in FCM profile.

AC: Asymptomatic HTLV-I carrier.

*P < 0.05 was regarded as statistical significant between Healthy PBMC and indicated category.

**Figure 4:**
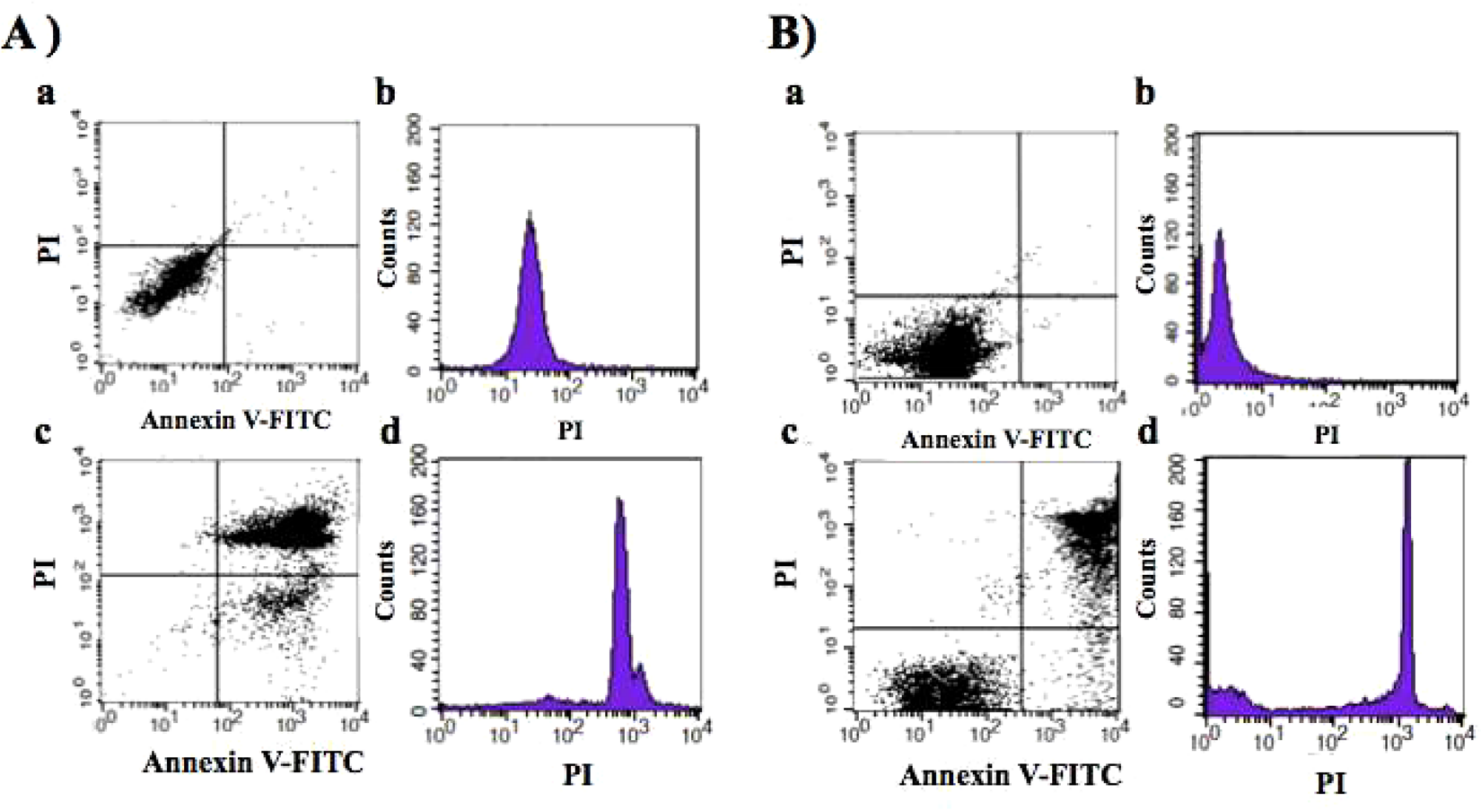
Apoptosis and/or necrosis induction using Photodynamic Therapy (PDT). PDT-induced apoptosis and/or necrosis in TLOm1 cells and chronic ATL PBMCs after incubation with 1 mM ALA for 24 h followed by 10 min light exposure which was detected by FCM using PI/Annexin V-FITC staining. (**A**) (a,b) TLOm1 cells cultured in 0 mM ALA followed by PDT showing survival of all cells. (c,d) Almost all the TLOm1 cells that were incubated in 1 mM ALA followed by PDT were killed via PI(−)/Annexin V(+) apoptosis or PI(+)/Annexin V(+) necrosis. (**B**) (a,b) Chronic ATL patient PBMCs surviving in 0 mM ALA-PDT. (c,d) Chronic ATL patient PBMCs were separated into PI(−)/Annexin V(−) alive or Annexin V(+) dead cells via necrosis or apoptosis after 1 mM ALA-PDT treatment.

Next, the TLOm1 cell line and chronic ATL patient PBMCs were exposed to light for 10 min using an Na-Li lamp after incubation with 1 mM ALA for 24 h, followed by FCM analyses using PI and Annexin V-FITC staining. TLOm1 cultured in the presence of 1 mM ALA showed 2 major populations of cells after light exposure treatment: Annexin V(+)/PI(−) apoptotic cells and Annexin V(+)/PI(+) necrotic cells, which indicated that almost all the cells died after PDT treatment. Contrastingly, 1 dominant Annexin V(−)/PI(−) live cell population was detected in the mock experiment containing 0 mM ALA followed by the same light exposure (Fig. 4A). The cells treated with 1 mM ALA without light exposure showed only one dominant Annexin V(−)/PI(−) live cell population. No apoptosis and/or necrosis cell population could be detected. PBMC specimens from chronic ATL patients subjected to the same treatment showed 2 dominant cell populations (AnnexinV(+)/PI(+) necrotic and Annexin V(−)/PI(−) alive) with a minor Annexin V(+)/PI(−) apoptotic population, indicating that their cells were divided into live and dead cell populations in contrast to the Annexin V(−)/PI(−) completely live cell population in the mock experiment (Fig. 4B).

**Figure 5:**
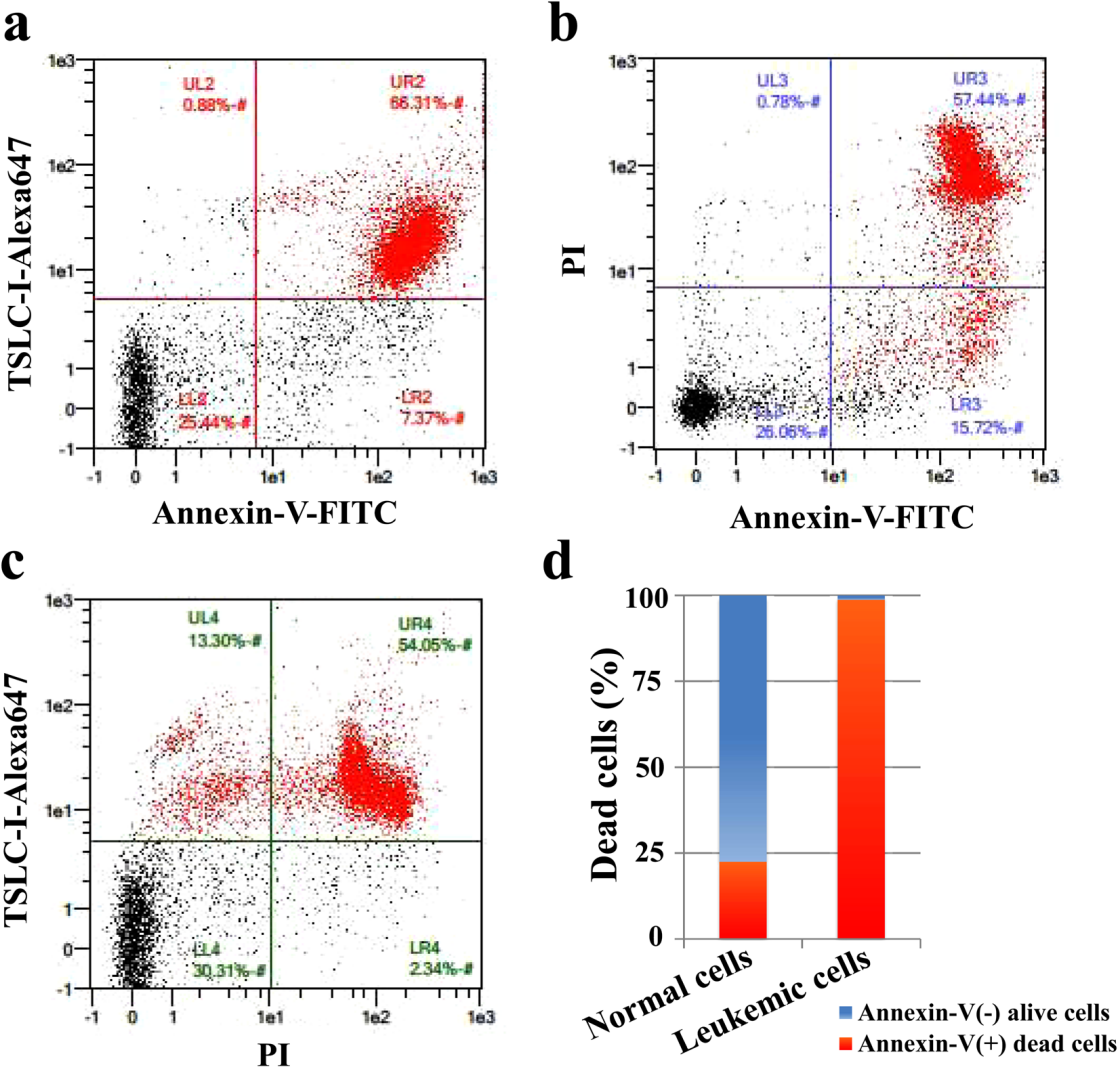
FCM analyses of ALA-PDT-treated chronic ATL patient PBMCs labeled with PI, TSLC1-Alexa647, and Annexin V-FITC. (**a**) TSLC1(+)/Annexin V(+) cells were observed to be dead ATL leukemic cells showing necrosis or apoptosis (red color); TSLC1(+)/Annexin V(−) cells were observed to be live ATL leukemic cells; TSLC1(−)/Annexin V(−) cells were observed to be live normal PBMCs; and TSLC1(−)/Annexin V(+) cells were observed to be dead normal PBMCs showing necrosis or apoptosis after ALA-PDT treatment. (**b**) PI(+)/Annexin V(+) cells were observed to be dead cells showing necrosis; PI(−)/Annexin V(+) cells were observed to be dead cells showing apoptosis; and PI(−)/Annexin V(−) cells were observed to be live cells. (**c**) TSLC1(−)/PI(+) cells were observed to be normal dead cells showing necrosis; TSLC1(−)/PI(−) cells were observed to be non-necrotic (live and/or apoptotic) normal cells; TSLC1(+)/PI(−) cells were observed to be non-necrotic (live and/or apoptotic) ATL cells; and TSLC1(+)/PI(+) cells were observed to be dead ATL leukemic cells showing necrosis. (**d**) Summary of FCM analyses showing that 98.7% of TSLC1(+) ATL leukemic cells were TSLC1(+)/Annexin V(+) dead cells (red), whereas 77.5% of TSLC1(−) normal cells were TSLC1(−)/Annexin V(−) live cells (blue) after ALA-PDT treatment, indicating that ALA-PDT induced highly-specific ATL leukemia cell death with minimal damage to normal PBMCs.

In order to clarify what kind of cell populations were alive or dead, 3-D FCM was performed using PI, Annexin V-FITC, and Alexa 647-anti-TSLC1 labeling, which showed that 98.7% of the TSLC1(+) ATL leukemic cell population consisted of Annexin V(+) dead cells. On the other hand, 77.5% of the TSLC1(−) normal cell population consisted of Annexin V(−) alive cells (Fig. 5).

**Figure 6:**
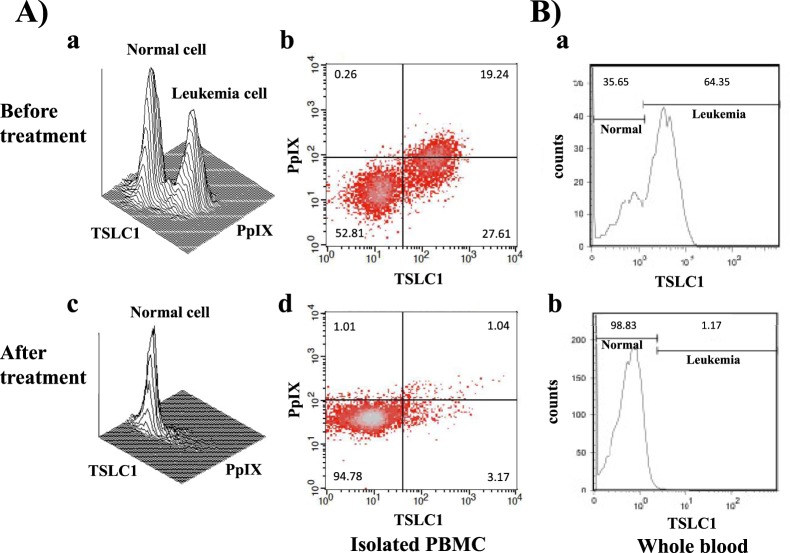
FCM analyses of chronic ATL patient specimens before and after ALA-PDT treatment of isolated PBMCs and whole blood. (**A**) (a,b) Chronic ATL PBMCs incubated in 1 mM ALA for 24 h showing 2 peaks indicating normal and ATL leukemic cells in TSLC1-FITC and PpIX profiles. (c,d) After 10 min of light exposure-treatment, the ATL leukemic cell peak completely disappeared and only the normal cell peak remained. (**B**) The total blood specimens of the chronic ATL patients were treated with ALA-PDT. (a) The TSLC1 profile showing normal and ATL leukemic cell peaks before PDT treatment. (b) After PDT treatment, the ATL leukemic cell peak disappeared and only the normal cell peak remained, which was essentially the same as the PBMC experiments.

To ensure that PDT specifically killed ATL leukemic cells, isolated PBMCs from chronic ATL patients were analyzed using the PpIX/TSLC1 profile of FCM, which showed 2 clear peaks that indicated normal and ATL leukemic cell populations without light exposure conditions (Fig. 6Aa,b). However, the ATL leukemic cell peak completely disappeared and only a single peak indicating normal cells was detected after light exposure (Fig. 6Ac,d). Whole blood PDT experiments using chronic ATL patient blood specimens essentially reproduced the same results as that of the PBMC experiments. However, prolonged light exposure times, ranging from 30–60 min, were needed.

**Figure 7:**
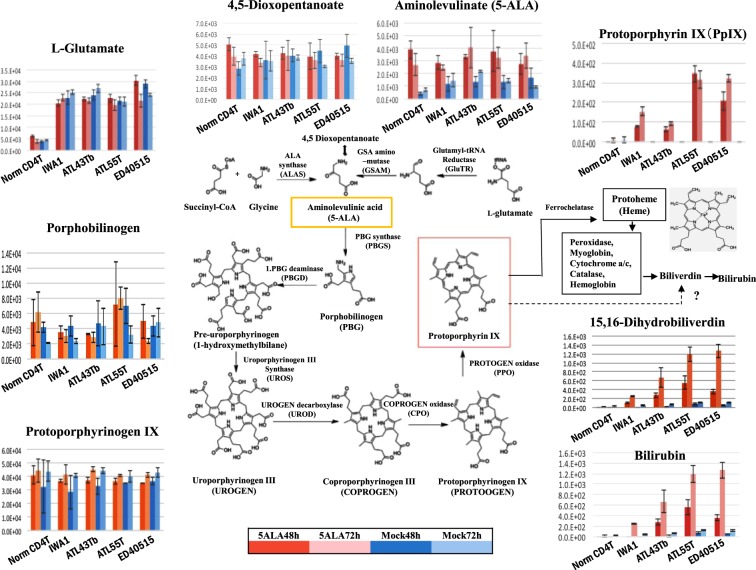
Metabolomics analyses of the porphyrin/heme synthetic pathway. Metabolomics analyses of the porphyrin/heme synthetic pathway in normal CD4(+) T cells, HTLV-1-immortalized cell line (IWA-1), and ATL leukemic cell lines (ATL43Tb, ATL-55T, and ED40515(−)) after incubation with (red bar) or without (blue bar) 1 mM ALA for 48 (thick bar) or 72 h (faint bar). Metabolites in the porphyrin/heme pathway, from ALA to protoporphyrinogen IX, did not show significant differences in the normal CD4(+) T cells, HTLV-1-immortalized cell line, and ATL leukemic cell lines. However, PpIX showed a striking increase from almost base-line in normal CD4(+) T cells to more than 10–100-fold in immortalized cells and ATL leukemic cells in the presence of 1 mM ALA for 48 or 72 h, respectively. An increase in the downstream metabolites, biliverdin and bilirubin, was also observed. 5-ALA was indicated in yellow box and PpIX was in red box. Bars indicate standard deviation (SD) of triplicate experiments.

In order to determine the metabolic steps that showed abnormal changes during the development and progression of ATL, we performed metabolome analyses using normal CD4(+) T cells, IWA-1 (an HTLV-I immortalized normal T cell line), and ATL leukemic cell lines (ATL43Tb, ATL-55T, and ED40515(−)). In the porphyrin and heme biosynthesis and catabolic pathways, no significant changes in ALA, porphobilinogen, uroporphyrinogen III, coproporphyrinogen III, and protoporphyrinogen IX were detected in the normal CD4(+) T cells, HTLV-I immortalized cells, and ATL leukemic cells in cultures with and without 1 mM ALA (Fig. 7). However, PpIX levels were dramatically different between them. PpIX levels in the ATL leukemic cells (ATL-55T and ED40515(−)) were more than several hundred-folds higher and in IWA-1 cells was 10–30-folds higher than that in the normal CD4(+) T cells when cultured in the presence of 1 mM ALA for 48 or 72 h. However, no accumulation of PpIX was found in any of these cells in the mock culture conditions without ALA. Moreover, downstream molecules of dihydrobiliverdin and bilirubin, showed similar profiles as that of PpIX.

## Discussion

For the dream of cancer treatment, not only killing the primary tumor, but also triggering the immune response simultaneously to recognize, chase after, and kill any remaining malignant cells at or near the site of the primary tumor and/or distant metastases would be hopeful. Most of the commonly used cancer treatments are immunosuppressive. Chemotherapy and X-irradiation treatments at the sufficient doses of destroy tumors are toxic to the bone marrow also, which is the source of all the cells of the immune system. Neutropenia and other forms of myelosuppression become evident with some other dose-limiting toxic side effects of these treatments. PDT has several potential advantages over surgery, chemotherapy and radiotherapy. PDT is comparatively non-invasive, can be targeted accurately, repeated doses can be applied without the total-dose limitations associated with chemotherapy and radiotherapy, and the moderate healing process results in little or no scarring. Furthermore, outpatients or day-case setting are usually possible for PDT treatments, which is convenient for the patients, and has no significant side effects^28,29^.

In the present study, we investigated the possibility of PDT in ATL. We found that PpIX accumulated 10–100-fold more in ATL leukemic cell lines and ATL patient leukemic cells when compared to the healthy fraction of PBMCs after a short-term culture in ALA, which enabled the highly-specific PDT-induced death of ATL leukemic cells with minimal damage to healthy PBMCs (Figs 4–6). Moreover, ATL leukemic cell lines (ATL43Tb, ATL-55T, and ED40515(−)) showed higher accumulation of PpIX when compared to ATL-derived non-leukemic cell lines (ATL16T, ATL6, and ED50823) and immortalized normal T-cell lines by HTLV-I infection (IWA-1, MT-4, and MT-2), suggesting the progression-dependent accumulation of PpIX from HTLV-I carriers to ATL non-leukemic and ATL leukemic states (Table S2). This phenomenon was further confirmed via metabolomics analyses, which showed the remarkable step by step accumulation of PpIX in normal CD4(+) T cells, HTLV-1 immortalized cells (a model for carriers), and ATL leukemic cells. These prominent, progression-dependent metabolomics changes were observed specifically at the step where ferrous irons were inserted into PpIX in the porphyrin-heme biosynthesis pathway, which is catalyzed by ferrochelatase (FECH)^28,29^. These changes were followed by downstream metabolites, biliverdin and bilirubin. No progression-dependent changes were observed in the intermediate metabolites from 5-ALA to protoporphyrinogen IX, which are adjacent precursor metabolites of PpIX. The PpIX accumulation phenomenon can be attributed to the dysregulation of FECH and/or ABC transporter G2 (ABCG2), which export PpIX from the mitochondria and cells and also act as multidrug resistance-associated proteins. This is consistent with previous findings^29–33^. In dormant cancer cells, transporter expressions of PEPT1, ALA importer, and ABCB6 (an intermediate porphyrin transporter) were upregulated and that of ABCG2 (a PpIX exporter) was downregulated^34^. The present metabolomics data indicated that PEPT1 and ABCB6 did not change enough to affect the porphyrin metabolic processes during the onset and progression of ATL. PpIX accumulation after ALA treatment was observed in several hematopoietic malignancies such as T-cell acute lymphoblastic leukemia (T-LBL), B-cell acute lymphoblastic leukemia (B-LBL), hairy cell leukemia, Burkitt’s lymphoma, follicular lymphoma, chronic myelogenous leukemia, and promyelocytic leukemia, in addition to other cancers and sarcomas (Table S2; Figs S1 and S2). Detailed investigations into the molecular dysregulation of FECH and ABCG2 in terms of progression-dependent PpIX accumulation may provide new insights into the pathogenesis of not only ATL, but also various kinds of malignancies.

TSLC1/CADM1, originally identified as a tumor suppressor in lung cancer, has recently been proposed to be a marker of malignant cells in ATL patients^35–37^. The aberrant expression of TSLC1 in ATL cells plays an important role in the growth and organ infiltration of ATL cells^36,37^. TSLC1 functions as a crucial scaffold molecule for Tax and Ubc13 to form a cellular complex with NEMO, TAX1BP1, and NRP in order to activate the IKK complex in the plasma membrane-associated lipid rafts, which in turn inactivates the NF-κB negative regulators and maintains persistent NF-κB activation in HTLV-1 infected cells^38^. TSLC1 expression is associated with the enhanced susceptibility of infected cells to cytotoxic T lymphocyte (CTL) lysis. Despite the immunodominance of Tax in CTL response, Tax(+)TSLC1(−) cells were inefficiently lysed by CTLs. High expression of TSLC1 in most HTLV-1-infected cells during enhanced CTL counter selection implies that TSLC1 confers a strong benefit on the virus^39^. FCM analyses, using independent makers for TSLC1/PpIX, detected the heterogeneities in chronic ATL patients and the details of the dynamically-changing patient statuses using the single cell resolution from ACs to smoldering-, chronic- and acute-type ATLs (Figs 3, S3 and S4). The LRI values clearly highlight these dynamically-changing ATL patient statuses and also enable the identification of the high-risk ACs in contrast to the HAM/TSP patients that exhibit no observable changes.

However, HAM/TSP patient specimens were found to have different FCM profiles, which showed an increase in PpIX(+)^dim^/TSLC1(−) and higher IRI values when compared to that of normal PBMCs. The signs and symptoms of HAM/TSP are caused by focal inflammatory lesions in the CNS. Inflammatory infiltrates of mononuclear cells can be widely dispersed throughout the CNS. The inflammatory process culminates, after months or years, in macroscopic changes in the CNS. HTLV-1 induces a Th1-like state in CD4(+)CCR4(+) T cells, which produces IFN-γ and an inflammatory positive feedback loop in HAM/TSP via astrocytes^40,41^. These evidences are consistent with the present findings. Dermatitis patient specimens also showed increased PpIX levels in addition to artificially-activated normal PBMCs after CD3/CD28 stimulation, suggesting that the PpIX increasing phenomenon may be common in activated cells. Although PpIX accumulation is much stronger in malignant cells when compared to those in non-malignant diseases, aberrantly-activated cells in non-malignant diseases. LRI and IRI values from the FCM analyses can be useful in characterizing the patient status based on the progressive abnormalities in the NF-κB and porphyrin-PpIX pathways of ATL and HAM/TSP.

PDT kills malignant tumor cells via apoptosis and necrosis and induces various effects in the tumor microenvironments, thereby affecting the tumor-associated or -infiltrating immune cells, which leads to the infiltration of various immune cells such as neutrophils and monocytes/macrophages into the targeted sites. This also stimulates the host immune system, causing acute inflammation to release various proinflammatory and acute-phase response mediators, including complement proteins, HSPs, arachidonic acid derivatives, chemokines, and cytokines such as TNF-α, IL-6, and IL-1^42–45^. PDT-treated dying cells produce danger signals called damage-associated molecular patterns (DAMPs), which increase antigen presentation by dendritic cells (DCs) and the recruitment of antigen-specific CTLs^42–46^. Acid ceramidase inhibitor, LCL521, also enhanced photodynamic therapy and photodynamic therapy-generated vaccine effects in order to effectively restrict the activity of Tregs and myeloid-derived suppressor cells (MDSCs)^47,48^. Antibodies against the immune checkpoint proteins, PD-1 and PD-L1, are novel therapeutic drugs for the treatment of cancers. ZnP@pyro PDT treatment combined with anti-PD-L1 results in the eradication of light-irradiated primary tumors and the complete inhibition of untreated distant tumors by generating a systemic tumor-specific cytotoxic T cell response^44,49^. Leukemia stem cells (LSCs) targeted by fluoroprobe indocyanine green (ICG)-loaded CPSNPs mediated PDT effectively reduced LSCs *in vitro* and also induced 29% disease-free survival in murine leukemia model *in vivo*^50^.

HTLV-1 infections cause ATL in 3–5% of HTLV-1 asymptomatic carriers (ACs) after a long latent period of 40–60 years^8,9^. Although about 95% of all ACs maintain the AC state without developing ATL throughout their lives, carriers always have to live in fear of possibly developing ATL. Dynamic changes of FCM analyses in Fig. 3 clearly identified the putative high risk ACs who show the similar FCM profile as that of smoldering ATL without symptom of ATL. On the other hand, some indolent ATL patients go on to develop blastic crisis, which signals progression to the aggressive form of ATL. Nearly half the number of aggressive ATL patients die within 6 months in spite of rigorous intervention^8,9,19^. Establishing a method that identifies high-risk indolent ATL patients who appear to have developed or are likely to develop the aggressive subtypes, and also establishing practical clinical treatments to prevent blastic crisis are challenges that need to be addressed urgently. HAS Flow method has been established, showing that the proportion of TSLC1^+^ subpopulations (TSLC1^pos^ CD7^dim^ and TSLC1^pos^ CD7^neg^) increased with the progression from AC to indolent and to aggressive ATL. This analysis identified the putative high risk AC and also the intermediate stage of ATL progression^51,52^. In the present study, ALA-PDT successfully induced 98.7% of ATL leukemic cell death with minimal damage on the healthy PBMCs in chronic ATL patient specimen, implying that ALA-PDT effectively inhibit the progression from indolent to aggressive ATL in addition to identification of intermediate stage of progression in ATL (Figs 5 and 6). As the poor penetration of light into tissues more than 3 mm thick in order to induce sufficient tumor necrosis and/or apoptosis, the ALA-PDT/PDD (Photodynamic therapy/photodynamic detection) system along with the circulatory system may be useful for the diagnosis and treatment of various types of malignancies like ATL and other types of leukemia, because of the direct correlation between the efficacy of PDT and higher accumulation of PpIX (Figs S1 and S2). Further research will be able to optimize many PDT-based parameters such as photosensitizer and light sources, photosensitizer and light doses, fluence rates, drug-light intervals, and combination with chemotherapy to increase the efficacy of the tumor-killing activity and also to stimulate the innate and adaptive anti-tumor immune responses.

## Materials and Methods

### Patients

PBMCs were isolated from the heparin-treated whole blood samples of healthy volunteers, HTLV-1 carriers, and patients with smoldering-, chronic-, acute-type ATL, and HAM/TSP via density gradient centrifugation using lymphocyte separation medium (LSM; Cosmo Bio, Tokyo, Japan). These patient specimens were collected at the Department of Pathology & Oncology, Graduate School of Medicine, Dentistry and Pharmaceutical Sciences, Okayama University in collaboration with the Okayama University Hospital, St. Marianna University School of Medicine and Imamura General Hospital. Informed consent was obtained from all the patients. The ATL diagnosis was based on clinical features, hematological characteristics, and monoclonal integrations of the HTLV-1 provirus determined via Southern blot analyses^6^. The HTLV-1 proviral DNA load was determined using real-time PCR as described previously^53^. Because both ATL and HAM/TSP are orphan diseases, all available patient samples were analyzed in this study. This study was approved by the Institutional Review Board at the Graduate School of Medicine, Dentistry and Pharmaceutical Sciences, Okayama University and related hospitals in accordance with the Declaration of Helsinki.

### Cell Culture

Culture conditions of the cell lines are summarized in Table S1. The cell lines ATL43Tb, ED40515(−), ATL16T, TLOm1, MT-1, HUT102, IWA-1, MT-4, MT-2, Jurkat, BALL1, Scott, Hair-M, Ramos, FL18, K562, and HL60 were maintained in RPMI-1640 medium supplemented with 10% fetal calf serum (FCS; Sankou Junyaku, Chiba, Japan), 100 U/mL of kanamycin (Meiji, Tokyo, Japan), and 100 μg/mL of streptomycin (LIFE Technologies, Rockville, MD, USA). ATL-55T, ED50823, and ATL6 were maintained in RPMI-1640 supplemented with 10% FCS, 100 U/mL of kanamycin, 100 μg/mL of streptomycin, and 20 U/mL of human recombinant IL2 (Strathmann Biotec GMBH, Hannover, Germany). A549, SCCKN, MCF-7, and HS-OS1 were grown in Dulbecco’s modified Eagle medium (Nissui Pharmaceuticals, Tokyo, Japan) supplemented with 5% FCS, 100 U/mL of kanamycin, and 100 μg/mL of streptomycin. PBMCs, isolated using the Ficoll-Hypaque method, were incubated in RPMI-1640 containing 10% FCS, 100 U/mL of kanamycin, and 100 μg/mL of streptomycin at 37 °C in a CO_2_ incubator. IWA-1 is an immortalized normal human T-cell line established by co-culturing with lethally X-ray-irradiated (10,000 R) MT-2 cells. The TLOm1 cell line, derived from an ATL patient, was supplied by Dr. Matsuoka M at Kyoto University; the ATL leukemic cell lines, ATL43Tb, ATL-55T, ED40515(−), and ATL-derived non-leukemic T-cell lines, ATL16T(−), ATL6(+), and ED50823(+), were supplied by Dr. Maeda M at Kyoto University; the HS-OS1 cell line was provided by Dr. Sonobe H at Okayama University; the FL18 cell line was provided by Dr. Oono at Kyoto University; the A549 cell line was obtained from the American Type Culture Collection (ATCC); the SCC-KN cell line was obtained from the RIKEN Cell Bank (Japan); the MCF-7 cell line was obtained from the Health Science Research Resources Bank (Japan); the Jurkat (established from T-cell acute lymphoblastic leukemia (T-LBL)), MT-2 and MT-4 (immortalized cell lines with the HTLV-I infection), BALL1, Scott, Hair-M, Ramos, K562, and HL60 cell lines were provided by Dr. Matsuo Y at the Fujisaki Cell Center, Hayashibara Biomedical Laboratories, Okayama, Japan. Normal CD4(+) T-cells were isolated from the PBMCs of healthy donors using the MACS human CD4(+) T-Cell Isolation Kit (Miltenyi Biotec, Bergisch Gladbach, Germany). The Dynabeads® Human T-Activator CD3/CD28 (Invitrogen, Oregon, USA) kit was used to activate and expand normal human T cells. The 5-ALA reagent was purchased from Cosmo Bio (Tokyo, Japan).

### Fluorescent microscopy

After incubation in 1 mM ALA for 24 h, the cells were diluted with PBS, centrifuged at 1,200 rpm for 5 min, and observed under a fluorescent microscope (Axiovert 200, Carl Zeiss Inc., Germany). Fluorescence images were taken using a highly light-sensitive, thermoelectrically-cooled charge-coupled device camera (Axiocam CCD camera, Zeiss). The filter combinations used included a 400-nm excitation filter, 580-nm beam splitter, and 590-nm long-pass emission filter for PpIX detection.

### Flow cytometry analysis of cellular PpIX and TSLC1

After incubating in the presence or absence of ALA, the cells were harvested via centrifugation at 1,200 rpm and 4 °C for 5 min, and washed with PBS. The cells were then suspended in 10% normal human serum and incubated for 15 min at 4 °C to prevent non-specific antibody binding. Next, the cells were labeled with chicken anti-TSLC1/CADM1 antibodies (MBL International Corporation, Code No. CM004-3, Clone:3E1, MA, USA), which were diluted in PBS containing 1% BSA, and incubated for 30 min on ice followed by washes with PBS containing 1% BSA. Alexa Fluor 488-Goat anti-Chicken IgY (Abcam, ab150169, Lot:GR227767, Cambridge, UK) was used as a secondary antibody, which was also incubated for 30 min on ice followed by washes with PBS. The cellular PpIX contents were measured using the FACSCalibur™ flow cytometer (BD Biosciences, San Jose, CA, USA) at an excitation wavelength of 488 nm and an emission wavelength of 650 nm, and quantified using the CellQuest™ software (BD Biosciences). A total of 10,000 cells were analyzed in each sample. Reproducibility was confirmed by experiments more than 3 times in cell line studies. Measurements of patient specimens were taken from distinct samples. Some patient PBMCs obtained were enough to measure repeatedly, reproducibility could be confirmed with repeated measurements.

### Experimental conditions for PDT

ALA was diluted in RPMI-1640 medium to make a 500-mM stock solution. The cells were incubated in a culture medium containing 0–1 mM ALA at 37 °C for 1–48 h under light shield conditions. The cells from the cell lines and PBMCs were then exposed to light for 10 min for the PDT experiments. For the whole blood experiments, fresh patient blood samples were exposed to light for 30–60 min after incubating with 1 mM ALA. An Na-Li lamp (TheraBeam VR630; USHIO Inc., Japan) was used as the light source along with a 37 °C water filter and a fan to prevent temperature increases in the specimens (Fig. 1A). The wavelength of the light used was 400–700 nm and the light intensity was 9.6 J/cm^2^. ALA-PDT-treated cells were labeled with chicken anti-TSLC1/CADM1 IgY, followed by labeling with Alexa 647-Donkey anti-Chicken IgY secondary antibodies (Millipore, Cat.#AP194SA6, Lot#Q2027093, CA, USA). The cells were then stained with Annexin V-FITC and propidium iodide (PI) using an Annexin V-FLUOS Staining kit (Roche Diagnostics, Mannheim, Germany) and PI obtained from Sigma Chemical Co. (St. Louis, MO, USA) according to the manufacturer’s instructions. Finally, the cells were analyzed using the MACSQuant^®^ Analyzer (Miltenyi Biotec, Bergisch Gladbach, Germany) and the data was analyzed using the FlowJo software (Treestar, San Carlos, CA, USA). For the whole blood PDT experiments, whole blood was circulated using the Perista^®^ Pump (ATTO Corp., Tokyo, Japan) during light exposure.

### Metabolomics analysis with LC-MS

For liquid chromatography-mass spectrometry (LC-MS), cell pellets (1 × 10^6^ cells/sample) were suspended in 200 µL of LC-MS grade methanol containing 100 mM L-ascorbic acid. Next, the cell suspensions were sonicated for 5 s using a probe-type sonicator, left on ice for 10 min, and centrifuged at 10,000 × *g* for 10 min at 4 °C. These preparations were done in the darkroom to prevent photobleaching of PpIX. The supernatants were stored at −80 °C until analysis.

Cell extracts (5 µL) were separated using an ODS column (L-column2 ODS, 1.5 × 150 mm, 3 µm; CERI, Japan) connected to an LC (Prominence, Shimadzu, Kyoto, Japan). The gradients used in mobile phase A and B (A: 0.1% formic acid in water, B: 0.1% formic acid in methanol for positive ion mode; A: 10 mM ammonium formate in water, B: 10 mM ammonium formate in 90% methanol for negative ion mode, respectively) was as follows: 0–3 min, 3% B; 3–16 min, 3–99% B; 16–45 min 99% B; 45–60 min, 0% B. The elute containing the separated molecules from the column was ionized with an ESI source heated to 250 °C, which works at 4.0 kV in positive ion mode or at 3.5 kV in negative ion mode. The sheath and auxiliary gas were set to 50 and 15 (arbitrary units), respectively.

The mass spectra (from *m/z* 50 to *m/z* 1,000) was measured using an Orbitrap Velos Pro Mass Spectrometer (Thermo Fisher Scientific, USA) in full scan mode. The ‘Lock mass’ function was enabled in order to retain the mass accuracy of the spectra, thereby detecting continuous peaks from environmental origins. For the positive ion mode, *m/z* 391.2843 [M + H]^+^ of 2-ethylhexyl phthalate was used and for the negative ion mode, *m/z* 255.2330 [M − H]^−^ of palmitic acid was used. The mass spectrometer was calibrated using poly-tyrosine ion peaks before analysis.

The LC-MS spectra was analyzed using the MZmine 2 software to perform peak extraction, data alignment with LC retention time, and normalization by total peak area^54^. Aligned peak lists were exported to a comma-separated value (CSV) format file and used for further statistical analysis.

### Statistical Analysis

Statistical analysis was performed by two-sided Student’s t-test to determine statistical significance between two groups using SPSS version24. For all tests, probability values of *P* < 0.05 were regarded as statistically significant.

## Electronic supplementary material

Supplemental information
