# Supplementary material for: Metabolic abnormalities in adult T-cell leukemia/lymphoma and induction of specific leukemic cell death using photodynamic therapy
Source: Sci Rep. 2018 Oct 8;8:14979. doi: 10.1038/s41598-018-33175-7 (PMC6175925; doi:10.1038/s41598-018-33175-7)
Supplement: Supplementary file 1 — Supplemental information [file 41598_2018_33175_MOESM1_ESM.pdf]

Supplementary information

Metabolic abnormalities in adult T-cell leukemia/lymphoma and induction of specific leukemic cell death using photodynamic therapy

Takashi Oka[1,2,*], Hajime Mizuno[3,#], Masumi Sakata[3], Hirofumi Fujita[4], Tadashi Yoshino[1], Yoshihisa Yamano[5], Kozo Utsumi[4], Tsutomu Masujima[3], and Atae Utsunomiya[6]

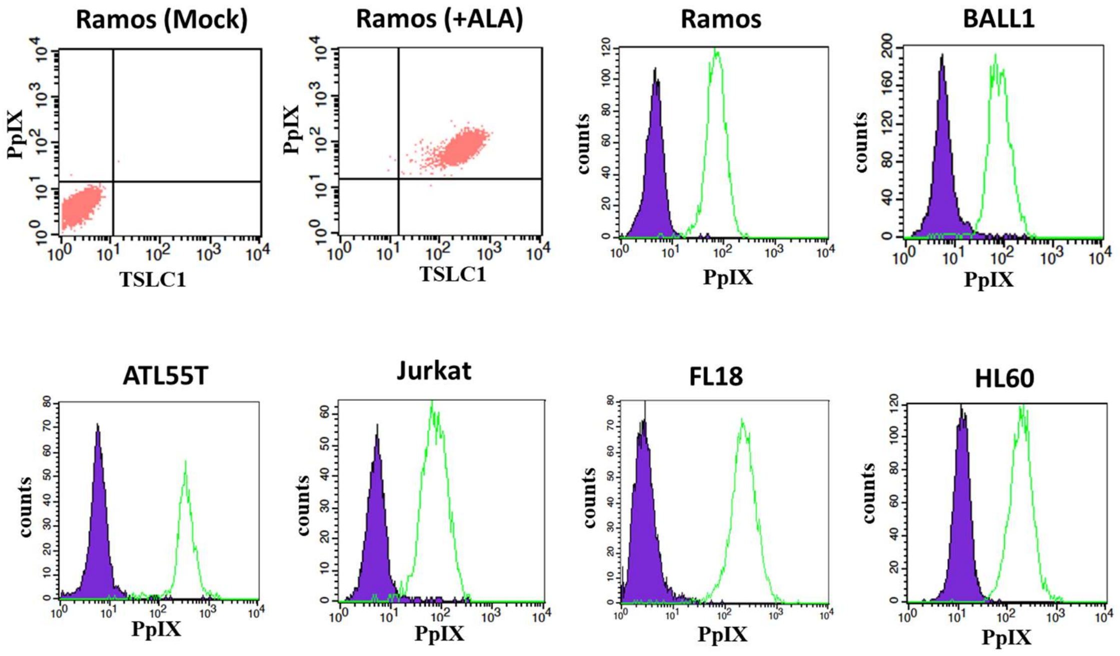

Figure S1. Aberrant accumulation of PpIX in hematopoietic malignant cell lines. Various T- and B-cell leukemia/lymphoma cells, myeloid leukemia cells, and ATL cells showing aberrant PpIX accumulation after incubation with 1 mM ALA for 24 h. ATL leukemic cell, ATL-55T; T-cell acute lymphoblastic leukemia, Jurkat; B-cell acute lymphoblastic leukemia, BALL1; Burkitt's lymphoma, Ramos; follicular lymphoma, FL18; and promyelocytic leukemia, HL60 cells.

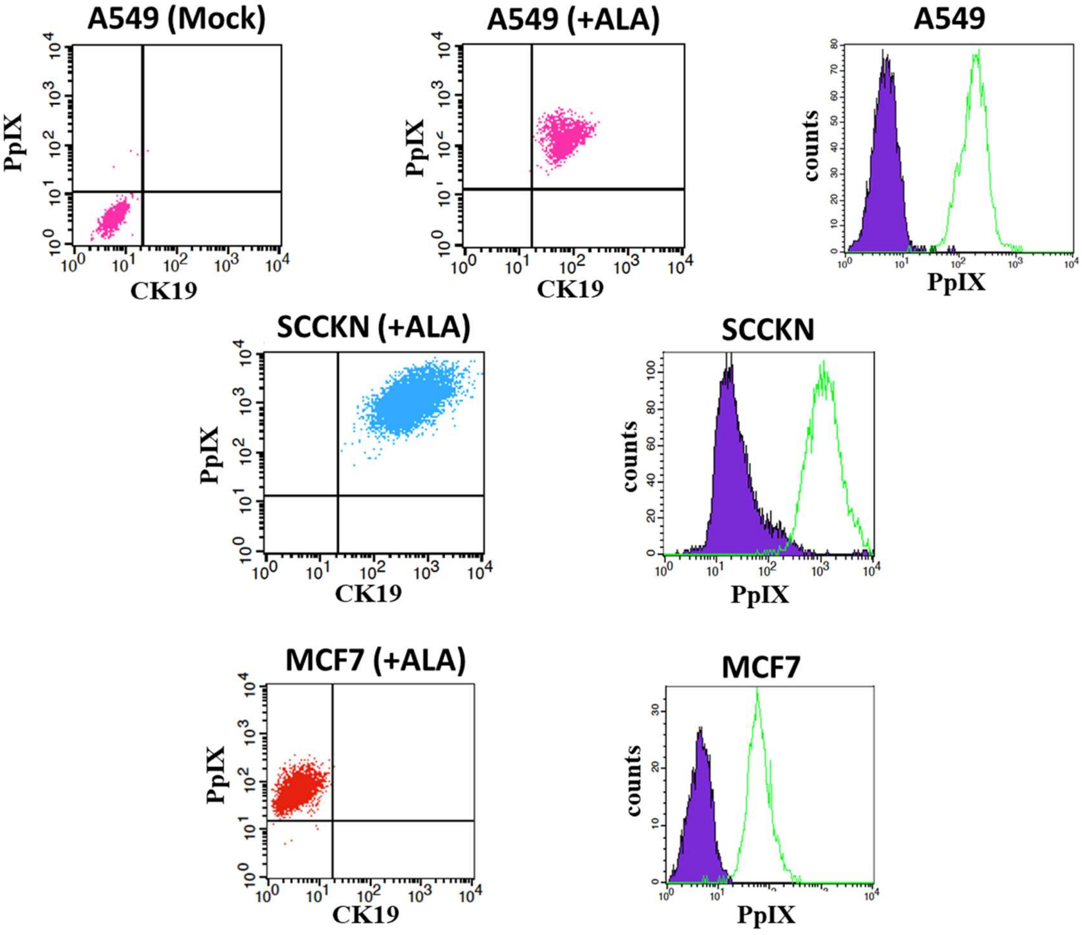

Figure S2. Aberrant accumulation of PpIX in cancer cell lines. FCM profiles of cancer cell lines using the PpIX/CK (cytokeratin)19 parameter. The lung cancer cell lines (A549), tongue cancer cell line (SCCKN), and breast cancer cell line (MCF-7) showed aberrant accumulation of PpIX after incubation with 1 mM ALA for 24 h.

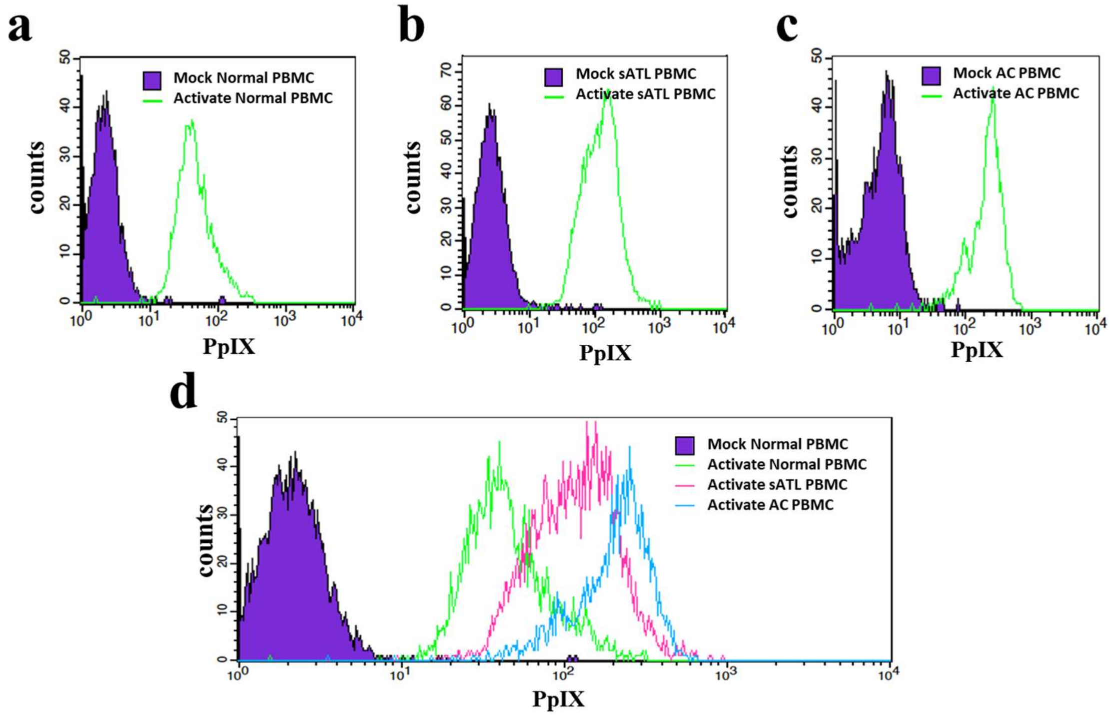

Figure S3. **Increased accumulation of PpIX after CD3/CD28 co-stimulation in normal PBMCs, ACs, and smoldering ATLs.** (**b, c, d**) Activated PBMCs from ACs and smoldering ATLs showing stronger accumulation of PpIX after CD3/CD28 co-stimulation and expanded culture for 2 weeks when compared to those of normal PBMCs (**a, d**), suggesting that aberrant metabolic changes, induced by HTLV-1 infection and/or onset of smoldering ATL, enhanced PpIX accumulation in response to 1mM ALA-treatment after co-stimulation with CD3/CD28.

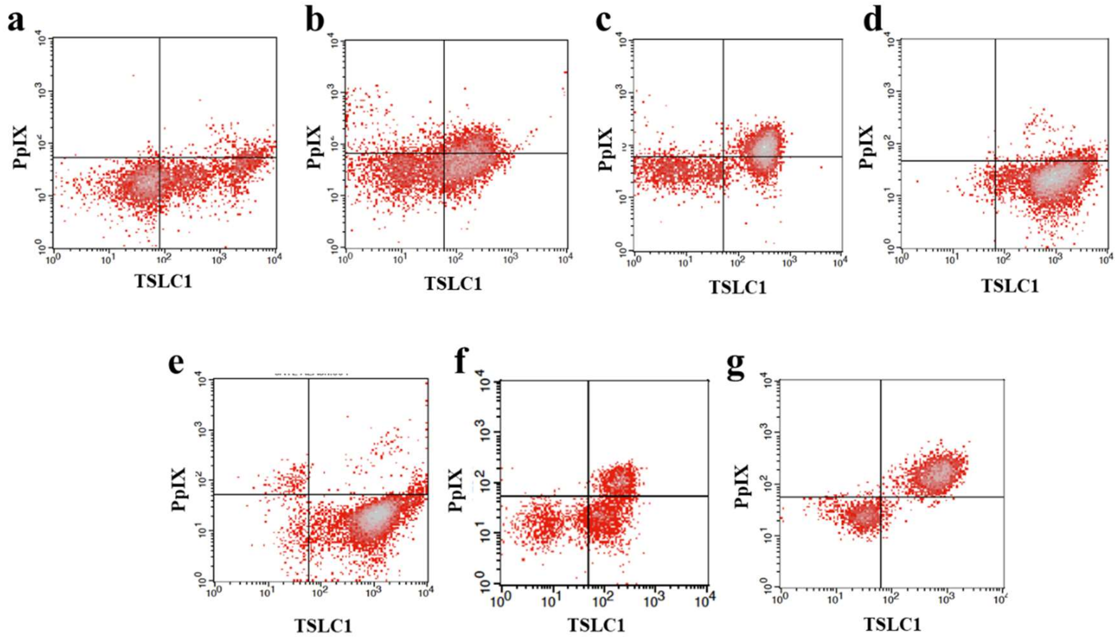

Figure S4. Heterogeneities in the PpIX/TSLC1 profile in chronic ATL patient PBMCs. There are various intermediate patterns in the PpIX-porphyrin metabolomic and TSLC1-related signal transduction pathways in chronic ATL patients based on the extent of abnormalities. (**a**) PpIX/TSLC1 profile showing 3 major cells populations: PpIX(-)/TSLC1(-), PpIX(-)/TSLC1(+)^{dim}, and PpIX(+)^{dim}/TSLC1(+)^{high}. (**b**) PpIX/TSLC1 profile showing 2 cell populations: PpIX(-)/TSLC1(-) and PpIX(+)^{dim} /TSLC1(+)^{dim}. (**c**) PpIX/TSLC1 profile showing 2 major cell populations: PpIX(-)/TSLC1(-) and PpIX(+)^{med}/TSLC1(+)^{med}. (**d**) PpIX/TSLC1 profile showing 2 major cell populations: PpIX(-)/TSLC1(+)^{dim} and PpIX(+)^{dim} /TSLC1(+)$^{med/high}$ with a minor population of PpIX(-)/TSLC1(-) cells. (**e**) PpIX/TSLC1 profile showing 1 dominant cell population (PpIX(-)/TSLC1(+)^{med}) with a minor populations of PpIX(-)/TSLC1(-), PpIX(-)/TSLC1(+)^{dim}, PpIX(+)/TSLC1(-), and PpIX(+)^{high} /TSLC1(+)^{high} cells. (**f**) PpIX/TSLC1 profile showing 3 major cell populations: PpIX(-)/TSLC1(-), PpIX(-)/TSLC1(+)^{dim}, and PpIX(+)^{med} /TSLC1(+)^{med}. (**g**) PpIX/TSLC1 profile showing 2 major cell populations: PpIX(-)/TSLC1(-) and PpIX(+)^{high} /TSLC1(+)$^{med /high}$.

Table S1. Culture condition and origin of cell lines.

Cell origin	Cell line name	Culture condition
ATL leukemic T-cell	ED40515	RPMI1640, 10%FCS
	ATL43Tb	RPMI1640, 10%FCS
	ATL55T	RPMI1640, 10%FCS, 20u/ml IL2
ATL-derived non-leukemic T-cell	ED50823	RPMI1640, 10%FCS, 20u/ml IL2
	ATL6	RPMI1640, 10%FCS, 20u/ml IL2
	ATL16T	RPMI1640, 10%FCS
ATL derived T-cell	TLOm1	RPMI1640, 10%FCS
	MT1	RPMI1640, 11%FCS
	HUT102	RPMI1640, 12%FCS
Immotalized normal T-cell by HTLV-I	IWA1	RPMI1640, 10%FCS
	MT4	RPMI1640, 10%FCS
	MT2	RPMI1640, 10%FCS
T-cell acute lymphoblastic leukemia (T-LBL)	Jurkat	RPMI1640, 10%FCS
B-cell acute lymphoblastic leukemia (B-LBL)	BALL1	RPMI1640, 10%FCS
B-cell acute lymphoblastic leukemia (B-LBL)	Scott	RPMI1640, 10%FCS
Hairy cell leukemia (HL)	Hair M	RPMI1640, 10%FCS
Burkitt Lymphoma (BL)	Ramos	RPMI1640, 10%FCS
Follicular lymphoma (FL)	FL18	RPMI1640, 10%FCS
Chronic myelogenous leukaemia (CML)	K562	RPMI1640, 10%FCS
Promyelocytic leukemia (PML)	HL60	RPMI1640, 10%FCS
Lung cancer	A549	DMEM, 5%FCS
Tongue cancer	SCCKN	DMEM, 5%FCS
Breast cancer	MCF7	DMEM, 5%FCS
Osteosarcoma	HS-OS1	DMEM, 5%FCS

HTLV-1: Human T-lymphotropic virus type1

Table S2. PpIX accumulation after incubation in the presence of 1mM ALA vs 0mM ALA(mock) in various kinds of cell lines

cell line	origin	Geo Mean (ALA)	Geo Mean (mock)	GeoMean(ALA)/GeoMean (mock)*
ATL55T	ATL leukemic T-cell	151.96	3.58	42.45
ATL43Tb	ATL leukemic T-cell	94.29	3.89	24.24
ED40515	ATL leukemic T-cell	164.93	7.76	21.25
TLOm1	ATL derived T-cell	587.12	3.78	155.32
MT1	ATL derived T-cell	119.19	4.08	29.21
HUT102	ATL derived T-cell	54.37	6.77	8.03
ATL16	ATL-derived non-leukemic T-cell	105.4	5.36	19.66
ATL6	ATL-derived non-leukemic T-cell	16.09	4.24	3.79
ED50823	ATL-derived non-leukemic T-cell	66.54	10.87	6.12
IWA1	Immotalized normal T-cell by HTLV-I	83.97	3.17	26.49
MT4	Immotalized normal T-cell by HTLV-I	41.98	2.91	14.43
MT2	Immotalized normal T-cell by HTLV-I	78.81	16.49	4.78
Jurkat	T-cell acute lymphoblastic leukemia (T-LBL)	46.67	3.49	13.37
BALL1	B-cell acute lymphoblastic leukemia (B-LBL)	79.79	4.52	17.65
Scott	B-cell acute lymphoblastic leukemia (B-LBL)	305.74	5.97	51.21
Hair M	Hairy cell leukemia (HL)	667.71	17.52	38.11
Ramos	Burkitt Lymphoma (BL)	69.63	4.44	15.68
FL18	Follicular lymphoma (FL)	222.61	2.82	78.94
K562	Chronic myelogenous leukaemia (CML)	99.5	15.85	6.28
HL60	Promyelocytic leukemia (PML)	180.95	11.85	15.27
A549	Lung cancer	131.08	3.45	37.99
SCCKN	Tongue cancer	1055.39	22.21	47.52
MCF7	Breast cancer	60.01	4.32	13.89
HS-OS1	Osteosarcoma	51.46	10.01	5.14

*: PpIX fluorescence intensity ratio of the cells, which were cultured in the presence/absence of 1mM ALA for 24 hrs. PpIX fluorescence intensity was measured by FCM.
